# Characterization of two transketolases encoded on the chromosome and the plasmid pBM19 of the facultative ribulose monophosphate cycle methylotroph *Bacillus methanolicus*

**DOI:** 10.1186/1471-2180-14-7

**Published:** 2014-01-09

**Authors:** Benno Markert, Jessica Stolzenberger, Trygve Brautaset, Volker F Wendisch

**Affiliations:** 1 Genetics of Prokaryotes, Faculty of Biology & CeBiTec, Bielefeld University, Universitätsstr. 25, 33615 Bielefeld, Germany; 2 Department of Molecular Biology, SINTEF Materials and Chemistry, Sem Selands vei 2A, 7465, Trondheim, Norway

**Keywords:** *Bacillus methanolicus*, Methylotrophy, Ribulose monophosphate (RuMP) pathway, Transketolase (TKT), Thiamine pyrophosphate (THDP) dependent enzyme

## Abstract

**Background:**

Transketolase (TKT) is a key enzyme of the pentose phosphate pathway (PPP), the Calvin cycle and the ribulose monophosphate (RuMP) cycle. *Bacillus methanolicus* is a facultative RuMP pathway methylotroph. *B. methanolicus* MGA3 harbors two genes putatively coding for TKTs; one located on the chromosome (*tkt*^*C*^) and one located on the natural occurring plasmid pBM19 (*tkt*^*P*^).

**Results:**

Both enzymes were produced in recombinant *Escherichia coli,* purified and shown to share similar biochemical parameters in vitro. They were found to be active as homotetramers and require thiamine pyrophosphate for catalytic activity. The inactive apoform of the TKTs, yielded by dialysis against buffer containing 10 mM EDTA, could be reconstituted most efficiently with Mn^2+^ and Mg^2+^. Both TKTs were thermo stable at physiological temperature (up to 65°C) with the highest activity at neutral pH. Ni^2+^, ATP and ADP significantly inhibited activity of both TKTs. Unlike the recently characterized RuMP pathway enzymes fructose 1,6-bisphosphate aldolase (FBA) and fructose 1,6-bisphosphatase/sedoheptulose 1,7-bisphosphatase (FBPase/SBPase) from *B. methanolicus* MGA3, both TKTs exhibited similar kinetic parameters although they only share 76% identical amino acids. The kinetic parameters were determined for the reaction with the substrates xylulose 5-phosphate (TKT^C^: k_cat_/K_M_: 264 s^-1^ mM^-1^; TKT^P^: k_cat_/K_M_: 231 s^-1^ mM) and ribulose 5-phosphate (TKT^C^: k_cat_/K_M_: 109 s^-1^ mM; TKT^P^: k_cat_/K_M_: 84 s^-1^ mM) as well as for the reaction with the substrates glyceraldehyde 3-phosphate (TKT^C^: k_cat_/K_M_: 108 s^-1^ mM; TKT^P^: k_cat_/K_M_: 71 s^-1^ mM) and fructose 6-phosphate (TKT^C^ k_cat_/K_M_: 115 s^-1^ mM; TKT^P^: k_cat_/K_M_: 448 s^-1^ mM).

**Conclusions:**

Based on the kinetic parameters no major TKT of *B. methanolicus* could be determined. Increased expression of *tkt*^*P*^, but not of *tkt*^*C*^ during growth with methanol [J Bacteriol 188:3063–3072, 2006] argues for TKT^P^ being the major TKT relevant in the RuMP pathway. Neither TKT exhibited activity as dihydroxyacetone synthase, as found in methylotrophic yeast, or as the evolutionary related 1-deoxyxylulose-5-phosphate synthase. The biological significance of the two TKTs for *B. methanolicus* methylotrophy is discussed.

## Background

Transketolase (TKT, EC 2.2.1.1) catalyzes the cleavage of a carbon-carbon bond adjacent to a carbonyl group in ketosugars and transfers a two-carbon ketol group to aldosugars [1,2], a reaction that might already have occurred under prebiotic conditions [3]. TKT requires divalent cations and thiamine diphosphate (ThDP) as a cofactor for its activity [4]. TKT is a key enzyme of the non-oxidative branch of the pentose phosphate pathway (PPP), the Calvin cycle and the ribulose monophosphate (RuMP) cycle. In these metabolic pathways, two ketol group transfers are relevant, the interconversion of xylulose 5-phosphate (X5-P) and ribose 5-phosphate (R5-P) to sedoheptulose 7-phosphate (S7-P) and glyceraldehyde phosphate (GAP) and the interconversion of GAP and fructose 6-phosphate (F6-P) to erythrose 4-phosphate (E4-P) and X5-P [5]. These substrates of TKT are important as precursors e.g. for nucleotide biosynthesis (R5-P), biosynthesis of aromatic amino acids (E4-P) and for cell wall biosynthesis in Gram-negative bacteria (S7-P). They are also intermediates of central pathways of carbon metabolism e.g. glycolysis (F6-P and GAP) and the Calvin and RuMP pathways [6].

TKT occurs in animals, plants, yeasts, archaea and bacteria like *Corynebacterium glutamicum*[7]. Properties of purified TKT have been reported mostly for eukaryotes like baker’s yeast [4], spinach [8], rat liver [9], mouse brain [10], human leukocytes/erythrocytes [11] but also from bacteria such *Escherichia coli*[12]. TKT is usually a homodimer with two active centers located at the interface between the contacting monomers. Methylotrophic yeasts possess a related enzyme, dihydroxyacetone synthases (DHAS, EC 2.2.1.3), which catalyzes the two-carbon ketol transfer from X5-P to formaldehyde yielding dihydroxyacetone phosphate (DHAP) and GAP. Thus, in these yeasts formaldehyde is assimilated by DHAS and the products DHAP and GAP are further metabolized to regenerate the X5-P and in other reactions of the central carbon metabolism [13]. DHAS has been purified from *Candida boidinii*[13] and from the carboxydobacterium *Acinetobacter* sp. [14] and is likely to be present in the actinomycete *Amycolatopsis methanolica*[15]. Besides DHAS and TKT also DHAS-like proteins have been described, but their function remains unknown [16].

The Gram-positive, thermotolerant and facultative methylotrophic bacterium *Bacillus methanolicus* that can use the one-carbon (C_1_) compound methanol as a source of carbon and energy [17-19] possesses two genes annotated to encode TKT [20]. One of them is encoded on the chromosome (*tkt*^*C*^), while the other one was found on the natural occurring plasmid pBM19 (*tkt*^*P*^) [20,21]. While the enzymes have not yet been characterized it has been proposed that they play an important role in the PPP and the RuMP pathway [20,22].

**Figure 1:**
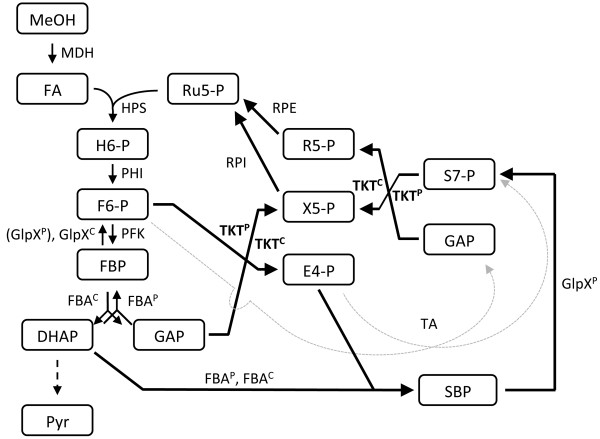
**Proposed map of the biochemical reactions of the methanol oxidation and assimilation pathways in *B. methanolicus *including the TA (dashed arrows) and the SBPase (solid arrows) variants of the RuMP pathway.** Enzymes: MDH*,* methanol dehydrogenase (EC 1.1.1.244); HPS*,* 3-hexulose-6-phosphate synthase (EC 4.1.2.43); PHI*,* 6-phospho-3-hexuloisomerase (EC 5.3.1.27); PFK*,* 6-phosphofructokinase, (EC 2.7.1.11); FBA*,* fructose-bisphosphate aldolase (EC 4.1.2.13); TKT*,* transketolase (EC 2.2.1.1); GlpX*,* fructose-bisphosphatase (EC 3.1.3.1); TA*,* transaldolase (EC 2.2.1.2); RPE*,* ribulose- phosphate 3-epimerase (EC 5.1.3.1); RPI*,* ribose-5-phosphate isomerase (EC 5.3.1.6); Metabolites: H6-P, 3-hexulose 6-phosphate; F6-P, fructose-6-phosphate; FBP, fructose-1,6-bisphosphate; GAP, glyceraldehyde 3-phosphate; DHAP, dihydroxyacetone phosphate; E4-P, erythrose 4-phosphate; SBP, sedoheptulose 1,7-bisphosphate; S7-P, sedoheptulose-7-phosphate; Ri5-P, ribose 5-phosphate; X5P, xylulose 5-phosphate; Ru5P, ribulose 5-phosphate; The reactions are described in detail in the text. Adapted from [28].

The initial reaction of methanol utilization in *B. methanolicus* is the oxidation of methanol to formaldehyde catalyzed by methanol dehydrogenase (MDH) [18]. It is known that *B. methanolicus* possesses three distinct active MDHs [23]. Reduction equivalents are generated by the linear dissimilation pathway of formaldehyde to CO_2_ and also by the PPP [24,25]. However, no formaldehyde dehydrogenase (FADH) was found in *B. methanolicus*[21]. Formaldehyde assimilation in *B. methanolicus* occurs via the RuMP pathway, which is divided in three different parts: fixation, cleavage and regeneration. The key reactions of the RuMP cycle are the aldol condensation of formaldehyde with ribulose 5-phosphate by 3-hexulose-6-phosphate synthase (HPS) and the subsequent isomerization of the product, D-arabino-3-hexulose 6-phosphate, to fructose 6-phosphate by 6-phospho-3-hexuloisomerase (PHI) in the fixation part. Fructose 1,6-bisphosphate (FBP) is generated in the subsequent phosphofructokinase reaction (Figure 1). Fructose 1,6-bisphosphate aldolase (FBA, EC 4.1.2.13) cleaves FBP into GAP and DHAP. *B. methanolicus* has one chromosomal- and one plasmid-encoded FBA (FBA^P^ and FBA^C^, respectively). Both catalyze the reversible cleavage of FBP to the triose phosphates GAP and DHAP [26]. We recently showed that FBA^P^ is presumably the major gluconeogenic FBA while FBA^C^ is the major glycolytic FBA in this bacterium [26]. The regeneration phase of the RuMP pathway, in which Ru5-P is regenerated, shares enzymes with the PPP and glycolysis [27] (Figure 1). Two different variants of the regeneration part of the RuMP pathway are known, the TA (transaldolase) variant and the SBPase (sedoheptulose 1,7-bisphosphatase) variant. Based on its genome sequence, *B. methanolicus* possesses the whole genetic equipment for both variants of the RuMP pathway [20-22]. In the TA variant, S7-P is directly generated from F6-P and E4-P by a TA activity, while the SBPase variant requires the activity of a sedoheptulose 1,7-bisphosphate aldolase (SBA) to generate sedoheptulose 1,7-bisphosphate (SBP) and an SBPase activity for the further conversion of SBP to S7-P. We recently demonstrated, that both FBAs from *B. methanolicus* are promiscuous enzymes also active as SBA, while only the plasmid encoded GlpX^P^ was active as FBPase and SBPase, which indicates that the SBPase variant of the RuMP pathway might operate in this organism [28]. Three enzymes, transketolase (TKT), ribose 5-phosphate isomerase (RPI) and ribulose 5-phosphate 3-epimerase (RPE), are shared in both variants. In the RuMP pathway, the predicted function of the TKT(s) is identical to the PPP and Calvin cycle.

It has been shown that the natural plasmid pBM19 carries the key *mdh* gene and five genes with deduced roles in the RuMP pathway (*glpX*, *fba*, *tkt*, *pfk*, *rpe*). The absence of pBM19 results in the loss of the ability to grow on methanol and caused higher methanol tolerance and reduced formaldehyde tolerance levels in *B. methanolicus* cells [20]. Transcription levels of *mdh* and the five plasmid encoded RuMP pathway genes, as well as the chromosomal genes *hps* and *phi*, were increased during growth with methanol suggesting their importance for methylotrophy [21,22]. Notably, 15 fold higher mRNA *tkt*^*P*^ levels were found in methanol- as compared to mannitol-grown cells [21,22]. Methanol consumption by this organism involves the concerted recruitment of both plasmid and chromosomal genes, and this discovery represented the first documentation of plasmid dependent methylotrophy [20,22,29].

The plasmid**-**encoded enzymes characterized to date differ from their chromosomally encoded counterparts as e.g. the three MDH enzymes exhibit different biochemical and physical properties and their genes are regulated differently [23]. GlpX^C^ was shown to be the major FBPase of *B. methanolicus*, while GlpX^P^ also carries SBPase activity [28]. Both FBA^C^ and FBA^P^ are SBAs, but their kinetic parameters allowed to distinguish FBA^C^ as major glycolytic FBA and FBA^P^ as major gluconeogenic FBA [26]. The objective of this study was to characterize the role and enzymatic properties of the two TKTs from *B. methanolicus* to get further insight into the genetic and biochemical aspects of methylotrophy

## Results

### Bioinformatic analysis and phylogeny of the TKT^P^ and TKT^C^ from *B. methanolicus*

**Figure 2:**
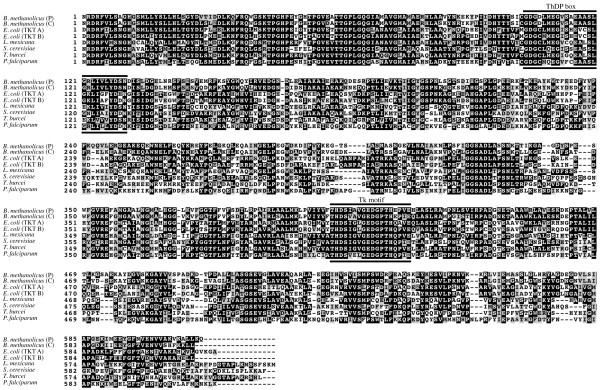
**Primary sequence alignment of TKT proteins.** Black and grey boxes indicate identical and similar residues. Bars indicate the characteristic ThDP motif and the TK motif. The sequence alignment was carried out using ClustalW, the alignment was formatted using BoxShade.

*B. methanolicus* possesses two distinct genes encoding TKT [21], *tkt*^*C*^ on the chromosome and the plasmid located *tkt*^*P*^. The deduced primary sequences of these proteins show a similarity of 87% (578/668) and an identity of 76% (506/668) to each other. The closest homolog of TKT^C^ present in the database is the chromosomally encoded homolog (EIJ77615.1; 97% identical amino acids) of *B. methanolicus* strain PB1. Similarly, the closest homolog of plasmid encoded TKT^P^ is the TKT (EIJ81398.1) from *B. methanolicus* PB1 (95% identical amino acids), which is encoded on plasmid pBM20. BLAST analyses of the amino acid sequences of TKT^C^ and TKT^P^ as queries suggested their classification as TKT with more than 200 sequences sharing 50% or more identical amino acids. An amino acid sequence alignment with biochemically characterized and experimentally verified TKTs from *E. coli* K12, encoded by *tktA* and *tktB*[12,30,31], *Plasmodium falciparum,* encoded by *pftk*[32], *Leishmania mexicana*, encoded by *tkt*[33], *Trypanosoma brucei*, encoded by *tbtkt*[34], and *Saccharomyces cerevisiae,* encoded by *sctk*[35] revealed the presence of highly conserved amino acid residues throughout the sequence with two notable motifs (Figure 2). The first N- terminal located motif is common to all Thiamindiphosphat (ThDP)-dependent enzymes. The sequence begins with the highly conserved residues Gly-Asp-Gly (GDG) followed by 21 less conserved residues [36,37]. The second so-called Tk motif is specific for all TKTs [38].

### Overexpression of *tkt*^*C*^ and *tkt*^*P*^ resulted in increased TKT activity in *B. methanolicus*

In order to study if the *tkt*^*C*^ and *tkt*^*P*^ genes encode functionally active TKT enzymes, both genes were overexpressed in *B. methanolicus*. Plasmids pTH1-*tkt*^*C*^ and pTH1-*tkt*^*P*^ were constructed based on pTH1 and with the *tkt* coding sequences under control of the methanol-inducible *mdh* promoter [20,39] and used to transform *B. methanolicus*. To confirm overexpression, TKT activities were determined in crude extracts of the resulting recombinant cells after growth in SOBSuc medium with or without 200 mM methanol. *B. methanolicus* carrying the empty vector pTH1 showed similar TKT activities regardless of the presence of the inducer (0.073 ± 0.004 U mg^-1^ under non-inducing conditions and of 0.075 ± 0.005 U mg^-1^ when methanol was present as inducer). When induced by methanol, the overexpression strains carrying either pTH1-*tkt*^*C*^ or pTH1-*tkt*^*P*^ showed significantly increased TKT activities of 0.373 ± 0.052 and 0.351 ± 0.064 U mg^-1^, respectively, as compared to non-inducing conditions (0.082 ± 0.002 and 0.083 ± 0.003 U mg^-1^, respectively). Thus, overexpression of *tkt*^*C*^ and *tkt*^*P*^ indeed increased transketolase activities 4–5 fold, confirming that both genes encode functionally active TKTs.

### Heterologous expression, purification and biochemical characterization of the TKT^P^ and TKT^C^

#### ***(I) Overexpression, purification and molecular mass detection***

The *tkt*^*P*^ and *tkt*^*C*^ coding regions were PCR-amplified and cloned into pET16b for production of the enzymes with an N-terminal His-tag (Table 1). The resulting plasmids were transformed into *E. coli* BL21 (DE3) and recombinant protein production was induced by the addition of IPTG to exponentially growing cell cultures. Cells were harvested, crude extracts were prepared and after Ni-NTA chromatography, His-tags were cleaved using factor Xa, and the enzymes were buffered in 50 mM Tris–HCl (pH 7.7). Protein purifications from 500 ml of culture broth led to average concentrations of about 1.2 mg/ml for both enzymes and a total amount of about 3 mg per purification.

**Table 1:** List of strains and plasmids used

**Strain, plasmid**	**Function and relevant characteristics**	**References**
***B. methanolicus***		
MGA3	Wild-type strain	[19]
***E. coli***		
DH5α	F^-^*thi-1 endA1 hsdR17(r*^*-*^ m^-^) *supE44 *ΔlacU169 (^-^80lacZΔM15) *recA1 gyrA96 relA1*	Bethesda research labs
BL21	*ompT hsdSB(rB*^*-*^ mB^_^) *gal dcm *(DE3)	Novagen [40]
**Plasmids**		
pEKEx3	Spe^R^; *C. glutamicum*/*E. coli *shuttle vector (*P*_*tac*_, *lacI*^q^; pBL1, *OriV*_*C.g.*_, *OriV*_*E.c.*_)	[41]
pHP13	*B. methanolicus*-*E. coli* shuttle vector; Clm^R^	[42]
pHP13mp	pHP13 carrying *lysC* coding region under control of the *mdh* promoter	[39]
pTH1mp-*lysC*	Similar as pHP13mp-*lysC* but with *Pci*I site upstream *mdh* promoter removed	[43]
pTH1mp	pTH1, but with a *mdh* promoter upstream to the mcs	This work
pTH1-*tkt*^*c*^ (Bme)	Derived from pTH1, for regulated expression of *tkt*^*c*^ of *B. methanolicus*	This work
pTH1-*tkt*^*p*^ (Bme)	Derived from pTH1, for regulated expression of *tkt*^*p*^ of *B. methanolicus*	This work
pET16b	Amp^R^; T7*lac*; vector for his-tagged protein overproduction	(Novagen)
pET16b-*tkt*^*c*^ (Bme)	For production of his-tagged TKT^C^ from *B. methanolicus*	This work
pET16b-*tkt*^*P*^(Bme)	For production of his-tagged TKT^P^ from *B. methanolicus*	This work
pET16b-*gapB*	Purification of his-tagged *E. coli* E4PDH from *E. coli* BL21(DE3)	This work

Abbreviations: Spe^R^, spectinomycin resistance; Clm^R^, chloramphenicol resistance; Amp^R^, ampicillin resistance.

Gel filtration of both proteins and TKT activity assays of the eluted fractions showed that both proteins eluted in a single fraction indicating that they are active as homotetramers with molecular weights for the tetramers of 280 kDa.

#### ***(II) Determining the optimal conditions for TKT activity***

The optimal assay conditions of the TKT enzymes were determined by using a coupled spectrometric assay for measuring the formation of GAP from R5-P and X5-P (as described in Materials and Methods). The activity of the auxiliary enzymes TPI and GPD were first checked under the different conditions and added in excess. Measurements were performed in 50 mM Tris–HCl buffer at 55°C and by using substrate concentrations of 1 mM for both TKT^C^ and TKT^P^, which is 7 and 5 times greater than the determined K_M_ values for TKT^C^ and TKT^P^, respectively (see below) Activity could be measured for both enzymes within a broad pH range between 6.5-10 for TKT^C^ and 5.5-9 for TKT^P^ with a pH optimum of pH 7.2-7.4 for both enzymes. All subsequent assays were performed at pH 7.5, the putative physiologically relevant pH.

The influence of the temperature, the pH, the effect of some metal ions and effectors were analyzed using enzyme Assay I (see materials and Methods). TKT activity in different buffers was tested and found to be almost independent of the buffer substance used in concentrations between 20 mM and 200 mM. Phosphate buffer, however, showed an inhibitory effect of the TKT activity of approximately 40%.

The highest activity of both TKTs was determined around 62°C, which corresponds roughly to the upper limit growth temperature of *B. methanolicus*. Temperatures higher than these resulted in strongly decreased TKT activities, which could be, to some extent, explained by the instability of the substrates triose phosphates [44] and/or reflect denaturation of the enzymes.

#### ***(III) TKT*** ***^C^*** ***displays higher temperature stability than TKT***^***P***^

The thermal stability of both TKTs was tested by pre-incubation of the proteins at temperatures ranging from 40 to 80°C. Samples were taken in different time periods and the activity was measured at 50°C under standard conditions. Both TKTs remained stable up to 50°C for at least 2 hours. Upon pre-incubation at 60°C the catalytic activity was reduced for both enzymes to approximately 60% within 10 minutes and then remained stable at this level. Incubation at 70°C led to a complete loss of activity for TKT^C^ after 4 minutes, for TKT^P^ after 30 minutes of incubation.

#### (IV) Formation of the TKT apoform and reconstitution of the holoenzyme revealed a bivalent metal ion dependency for activity

**Figure 3:**
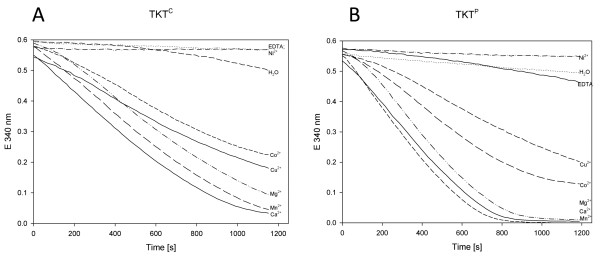
**Reconstitution of apoforms of TKT**^**C **^**(A) and TKT**^**P **^**(B) in the presence of different divalent cations.** The reaction was measured according to the enzyme assay I (Methods) with the standard substrates R5-P and X5-P and dialyzed TKT preparations. Each reaction mixture contained 1 mM divalent cations and 150 ng purified TKT enzyme. At t = 0, the assay was started by the addition of THDP to a final concentration of 20 μM. The decrease in absorbance at 340 nm as a result of NADH oxidation was monitored over time.

During optimization of the assay conditions for the TKT activity, a dependence of bivalent cation for both TKTs was observed. Therefore, the apo-TKT form was obtained for both *B. methanolicus* TKTs by removing any bound cofactors via dialysis for 24 hours against Tris–HCl buffer containing 10 mM EDTA. After EDTA was removed by subsequent dialysis, different divalent metal ions, including Co^2+^, Ni^2+^, Cu^2+^, Mn^2+^, Mg^2+^ and Ca^2+^ were tested as putative cofactors for both TKTs at a final concentration of 1 mM (Figure 3). Reconstitution of the TKT activity was stimulated by Mn^2+^, Mg^2+^, Co^2+^, Ca^2+^ and Cu^2+^. The addition of Ni^2+^ did not restore the TKT activity at all, while slow reconstitution was observed with water, presumably due to contamination of substrates or buffer components with divalent cations.

#### ***(V) TKT activities are inhibited by ATP, ADP, EDTA and Ni***^***2+***^

To identify inhibitors or activators of *B. methanolicus* TKT activity, potential effectors were tested at concentrations of 1 and 5 mM. TKT^P^ and TKT^C^ were both inhibited by ATP (65% and 75%, respectively) and by ADP (65% and 95%, respectively). EDTA in concentration of 10 mM resulted for both TKT in a completely loss of activity. Ni^2+^ at a concentration of 1 mM also led to a complete loss of activity for both TKT.

### TKT^P^ and TKT^C^ share similar kinetic parameters and substrate spectrum

The kinetic parameters of TKT^C^ and TKT^P^ were determined for the conversion of F6-P and GAP to X5-P and E4-P as well as for the formation of S7-P and GAP from X5-P and R5-P in vitro (Table 2). The assays were performed at 60°C and pH 7.5 in 50 mM Tris–HCl with 2 mM MnCl_2_ and 1 μM THDP. Both recombinant TKTs catalyzed the conversion of X5-P and R5-P to GAP and S7-P with comparable kinetic parameters. For X5-P and TKT^C^ a K_M_ of 150 μM ± 4 μM and a V_max_ of 34 ± 1 U/mg could be determined, whereas TKT^P^ displayed a K_M_ of 232 μM ± 2 μM and V_max_ of 45 ± 1 U/mg. Similar parameters could be measured for the second substrate R5-P, for which TKT^C^ has a K_M_ of 118 μM ± 13 μM and a V_max_ of 11 ± 1 U/mg, TKT^P^ shows a K_M_ of 250 μM ± 13 μM and V_max_ of 18 ± 1 U/mg. The catalytic efficiencies for both TKTs are accordingly quite similar for X5-P (for TKT^C^ 264 s^–1^ mM^–1^ and for TKT^P^ 231 s^–1^ mM^–1^) and this also holds for R5-P (for TKT^C^ 109 s^–1^ mM^–1^ and for TKT^P^ 84 s^–1^ mM^–1^). Comparable catalytic efficiencies could be calculated for GAP (for TKT^C^ 108 s^–1^ mM^–1^ and for TKT^P^ 71 s^–1^ mM^–1^) while for F6-P the catalytic efficiency for TKT^P^ is about 4-fold higher than that of TKT^C^ (448 s^–1^ mM^–1^ and 115 s^–1^ mM^–1^, respectively) Following affinities were observed for GAP (TKT^C^ K_M_ 0.92 ± .033 mM and a Vmax 85 ± 3 U/mg; TKT^P^ K_M_ 0.67 ± .012 mM and Vmax 42 ± 4 U/mg) and F6-P (TKT^C^ K_M_ 0.72 ± 0.11 mM and a Vmax of 71 ± 11 U/mg; TKT^P^: K_M_ 0.25 mM and Vmax 96 ± 5 U/mg).

**Table 2:** **Biochemical properties of TKT**^**P **^**and TKT**^**C**^

Parameter		**TKT**^**C**^	**TKT**^**P**^
Molecular weight		73 kDa	73 kDa
		280 kDa (tetramer)	280 kDa (tetramer)
Optimal activity conditions:		50 mM Tris–HCl, pH 7.5, 2 mM Mn^2+^, 2 μM THDP, 55°C	50 mM Tris–HCl, pH7.7, 5 mM Mn^2+^, 1 μM THDP, 55°C
Optimal pH		7.2-7.4	7.2-7.4
Optimal temperature		62°C	62°C
Temperature stability		< 60°C	< 60°C
**Kinetics**			
X5P	K_M_	0.15 ± 0.01 mM	0.23 ± 0.01 mM
	V_max_	34 ± 1 U/mg	45 ± 28 U/mg
	k_cat_	40 s^-1^	54 s^-1^
	k_cat_/K_M_	264 s^–1^ mM^–1^	231 s^–1^ mM^–1^
R5P	K_M_	0.12 ± 0.01 mM	0.25 ± 0.01 mM
	V_max_	11 ± 1 U/mg	18 ± 1 U/mg
	k_cat_	13 s^-1^	21 s^-1^
	k_cat_/K_M_	109 s^–1^ mM^–1^	84 s^–1^ mM^–1^
GAP	K_M_	0.92 ± 0.03 mM	0.67 ± 0.01 mM
	V_max_	85 ± 3 U/mg	42 ± 1 U/mg
	k_cat_	99 s^-1^	48 s^-1^
	k_cat_/K_M_	108 s^–1^ mM^–1^	71 s^–1^ mM^–1^
F6P	K_M_	0.72 ± 0.11 mM	0.25 ± 0.01 mM
	V_max_	71 ± 11 U/mg	96 ± 5 U/mg
	k_cat_	82 s^-1^	112 s^-1^
	k_cat_/K_M_	115 s^–1^ mM^–1^	448 s^–1^ mM^–1^

Values for K_M_ (mM), V_max_ (U/mg), and catalytic efficiency (k_cat_/K_M_ = s^-1^ mM^-1^) were determined for two independent protein purifications and mean values and arithmetric deviations from the mean are given.

The kinetics of the reverse reactions could not be determined since neither E4-P nor S7-P are currently available commercially. An additional activity as DHAS, as found in methylotrophic yeasts, or as the evolutionary related DXP synthase could not be observed.

## Discussion

The biochemical results provided here show that the plasmid (TKT^P^) and chromosomally (TKT^P^) encoded TKTs are similar and based on these data it is not feasible to predict their individual roles for methylotrophy in *B. methanolicus.* Both TKTs are active as homotetramers, a characterisitic shared with TKTs from *Triticum aestivum* and *Sus scrova*[5], but different from several microbial TKTs such as the enzymes from *E. coli*[12,45], *Saccharomyces cerevisiae*[46] and *Rhodobacer sphaeroides*[47]. The requirement of bivalent cations for the activity of TKT from *B. methanolicus* with a preference of Mn^2+^. Mg^2+^, and Ca^2+^ is a common feature of TKTs, while the efficiency for the cations varies between different TKTs [12,48]. It was assumed in the past, that purified mammalian TKTs do not require the addition of cofactors to maintain activity [9]. This led to the wrong conclusion that these enzymes did not require bivalent cations for activity. This was because the complex of TKT with THDP and cation is strong enough to carry the cofactors along the purification steps and though TKT remaining active. The cation can be removed by dialysis against EDTA [9,49,50]. Both TKTs showed comparable biochemical properties. This is in contrast to the recently characterized and biochemically diverse MDHs from *B. methanolicus*, which displayed different biochemical and regulatory properties [23]. Both TKTs were shown to be thermo stable at physiological temperature (50°C) of *B. methanolicus*. Neutral pH (6.5 to 7.8) was also reported to be optimal for both enzymes of *E. coli*[13,31] and *S. cerevisiae*[51] and *Rhodobacter sphaeroides*[47]*.* Inhibition by ATP and ADP is unusual, however, since the intracellular concentrations of ATP and ADP in *B. methanolicus* are not known, it is difficult to judge the relevance of this inhibition *in vivo.*

TKT has been found so far in all organisms that have been investigated [31]. The presence of more than one TKT however, as described here for *B. methanolicus* is not a common phenomenon. Two TKTs are known in *S. cerevisiae*, encoded by *tkl1* and *tkl2*[52,53], and *E. coli*, encoded by *tktA* and *tktB*[12,30]. As in *B. methanolicus*, the TKTs of *E. coli* and *S. cerevisiae* exhibit comparable kinetic parameters. However, deletion of *tkl1*, probably encoding the main TKT in *S. cerevisiae,* impaired growth in synthetic medium without added aromatic amino acids, whereas deletion of *tkl2* did not cause such phenotype. In *E. coli*, the *tktA* gene product is the major isoenzyme and accounts for about 70 to 90% of TKT activity in cells and *tktA* mutants are highly sensitive to the presence of D-ribose, while *tktB* deletion mutants are not. *tktA tktB* double mutants are viable, but deficient in pentose catabolism and they require the addition of all three aromatic amino acids, aromatic vitamins and pyridoxine (vitamin B6). Transketolase A from *Escherichia coli* was shown to derepress the multiple antibiotic resistance operon *marRAB* by binding to the repressor MarR [54]. It remains to be shown if the TKTs from *B. methanolicus* show regulatory interactions with transcriptional repressors and if TKT^P^ and TKT^C^ differ in this respect.

Besides the common sugar phosphates F6-P, R5-P, GAP, X5-P and E4-P, TKTs from spinach leaves and *S. cerevisiae* are able to also utilize DHAP, dihydroxyacetone (DHA) and HP [50,55,56]. The reaction of TKTs with formaldehyde (called DHAS) is known in methylotrophic yeasts [57] and was recently also reported for transketolase A of *E. coli*[31]. However, among all substrates tested, both TKTs form *B. methanolicus* were only active with X5-P and R5-P as well as F6-P and GAP. Similar substrate specificity was described for mammalian TKTs [58]. Based on the catalytic efficiency (TKT^C^ 82 s^–1^ mM^–1^ versus TKT^P^ 448 s^–1^ mM^–1^) TKT^P^ appears better suited for the interconversion of S7-P and GAP to R5-P and X5-P.

About 15 fold higher mRNA levels of *tktP*, but not of *tktC,* were previously observed when comparing growth in minimal medium with methanol and mannitol [21]. This induction was not observed here when assaying crude extracts of *B. methanolicus* MGA3(pTH1) which carries endogenous plasmid pBM19 after growth in complex medium SOBSuc induced with 200 mM methanol. Likely, this difference is due to the use of different media, namely complex medium with methanol vs. methanol minimal medium.

## Conclusion

Both, TKT^P^ and TKT^C^, showed comparable kinetic parameters. The about 15 fold increased mRNA levels of *tkt*^*P*^ and of other RuMP pathway genes on the plasmid pBM19, which is essential for methanol utilization [12,21] during growth in methanol minimal medium as compared to growth in mannitol minimal medium [20] argues for TKT^P^ being the major TKT relevant in the RuMP pathway. In line with this argumentation, methanol-inducible GlpX^P^ carries SBPase activity, which is relevant in the RuMP pathway [28], while the chromosomally encoded GlpX^C^ is the major FBPase in gluconeogenesis and is not methanol-inducible.

## Methods

### Microorganisms and cultivation conditions

*B. methanolicus* strains were grown at 50°C in the following media. SOBsuc medium is SOB medium (Difco) supplemented with 0.25 M sucrose. Bacterial growth was performed in shake flasks (500 ml) in 100 ml medium at 200 r.p.m. and monitored by measuring the OD_600_. The inoculation of the precultures for all growth experiments of *B. methanolicus* strains was performed with frozen ampules of *B. methanolicus* as a starter culture. Ampules of *B. methanolicus* cells were prepared from exponentially growing cultures (OD_600_ 1.0 to 1.5) and stored at -80°C in 15 % (v/v) glycerol [22]. For inoculation, ampules were thawed and 250 μl cell suspension was used to inoculate 100 ml medium. The *E. coli* strain DH5α was used as a standard cloning host [59]. Recombinant cells were grown in lysogeny broth (LB) medium at 37°C supplemented with ampicillin (100 μg/ml), kanamycin (50 μg/ml), spectinomycin (100 μg/ml), and 1 mM IPTG when appropriate. Recombinant *E. coli* procedures were performed as described elsewhere [60]. Recombinant protein production was carried out with *E. coli* BL21 (DE3) as the host [61]. Bacterial strains and plasmids used in this work are listed in Table 1 and oligonucleotides for PCR and cloning are listed in Table 3.

**Table 3:** List of oligonucleotides used

**Name**	**Sequence (5’-3’)**
pET16b_Fw	GCTAACGCAGTCAGGCACCGTGTA
pET16b_Rv	GACTCACTATAGGGGAATTGTGAGCG
tktC_Fw_XhoI	CCGG**CTCGAG***TTG*TTTGATAAAATTGACCAT
tktC_Rv_XhoI	CCGG**CTCGAG***TTA*TTGTTTAAGTAAAGCT
tktP_Fw_XhoI	GCGC**CTCGAG***GTG*CTCCAACAAAAAATAGAT CG
tktP_Rv_XhoI	GGCG**CTCGAG***TTA*GAGAAGCTTTTTAAAATGAGAAA
tkt_C_Seq1	GCGTCATTTGGCAGCGGTATATAAT
tkt_C_Seq2	TCTAGGTCCTGAAGAACGAAAGC
tkt_C_Seq3	GGCTCGGCAGATCTTGCTAGTTC
tkt_P_Seq1	CCCTCATACGCTTTTTCAGAATC
tkt_P_Seq2	GCTAGAGCATTTAACACTGCACC
tkt_P_Seq3	CGATCTTGAACACTCTCACTAAATG
gapb_fw	GCGA**CTCGAG***ATG*ACCGTACGCGTAGCGATAA
gapb_rv	GCGT**CTCGAG***TTA*CCTGAAAGCAACAGTAGC

Restriction sites are highlighted in italics, stop and start codons are underlined.

### Homologous overexpression of *tkt*^C^ and *tkt*^*P*^ in *B. methanolicus*

Overexpression vector pTH1 was used to allow methanol inducible expression of *B. methanolicus* TKT genes. This vector is analogous to the plasmid pHP13, in which the strong *mdh* promoter was cloned in-frame with the *mdh* rbs region to allow methanol inducible expression in *B. methanolicus*[20,39]. The DNA fragments of the *tkt*^*C*^ and *tkt*^*P*^ coding regions were amplified from DNA of *B. methanolicus* by the primer pair *tkt*_P-Bme-fw and *tkt_*P-Bme-rv, and *tkt*_C-Bme-fw and *tkt*_C-Bme-fw (Table 3). The resulting PCR products were digested with *Pci*I and ligated to the *Pci*I digested vector pTH1. The resulting vectors were named pTH1-*tkt*^*C*^(Bme) and pTH1-*tkt*^*P*^(Bme), respectively*.* Crude cell extracts were prepared based on the protocol described elsewhere [20]. *B. methanolicus* cells were grown in SOB medium with 0.25 mM sucrose to stationary phase (OD_600_, 2.5 to 3.3). Gene expression was induced by addition of 200 mM methanol at inoculation. 20 ml of the cell culture was harvested by centrifugation (4000 × g, 10 min, 4°C), washed in 50 mM potassium phosphate buffer (pH 7.5) and stored at -20°C. The cells were disrupted by sonication described [29]. Cell debris was removed by centrifugation (14,000 x g, 1 h, 4°C) and the supernatant was collected as crude extract. TKT activity was measured according to assay II.

### Purification molecular mass determination of TKT proteins

For protein production with *E. coli* BL21 (DE3) [61], *tkt*^*P*^ and *tkt*^*C*^ were amplified by PCR using the primers *tkt*_C-Xho-fw and *tkt*_C-Xho-rv and *tkt*_P-Xho-fw and *tkt*_P-Xho-rv (Table 3). The resulting PCR products were ligated, after restriction with *Xho*I, into *Xho*I restricted pET16b (Novagen, Madison, Wisconsin, USA), resulting in pET16b-*tkt*^*C*^ and pET16b-*tkt*^*P*^. The pET16b vector allows the production of an N-terminal decahistidine tagged TKT in *E. coli* BL21 (DE3). Protein production and purification was performed as described previously [62]. Both enzymes were purified to homogenity. After purification, the His-tag was cleaved by factor Xa (Novagen, San Diego) according to the manufacturer’s recommendations and buffered in 20 mM Tricine, pH 7.7. The protein purification was analyzed by 12% SDS-PAGE [63]. Protein concentration was measured according the method of Bradford using the Bio-Rad Protein-Assay with BSA as standard. The tetrameric structures of the TKT proteins were determined by gel filtration as described previously [62] using 1 mg TKT dissolved in 2 ml of 20 mM Tris–HCl, pH 7.5.

### Enzyme assays for the purified TKT proteins

The TKT activity in the direction of S7P + GAP from R5P + Xu5P was done by Assay I, a modified version of a previously described assay [31] using the auxiliary enzymes triose-phosphate isomerase (TPI) and glycerol 3-phosphate dehydrogenase (GPD) from rabbit muscle. The oxidation of NADH was followed setting 1 pmol NADH oxidized equivalent to 1 pmol X5-P consumed. The standard reaction mixture (final volume 1 ml) contained 50 mM Tris–HCl buffer (pH 7.5), 0.25 mM NADH, 2 mM Mn_2_Cl, 0.4 U/ml TPI, 0.7 U/ml glyceraldehyde-3-phosphate dehydrogenase (GAPDH) and purified TKT protein which was preheated for 3 min at 50°C. NADH reduction (ϵ_340nm_ = 6.22 mM^–1^ cm^–1^) was followed at 340 nm on a Shimadzu UV1700 spectrophotometer. The reaction was initiated by the addition of R5-P or X5-P, respectively (final concentration varied between 0.05 - 10 mM). The pH-optimum was defined by using the following buffers (50 mM): acetate (pH 5.0-6.0), phosphate (pH 6.0-7.0), Tris–HCl (pH 7.0-9.0), and glycine-NaOH (pH 9.0-10.0) under standard conditions. The pH was adjusted at 50°C.

### Formation of the transketolase apoform and reconstitution of the holoenzyme

Apo-transketolase was obtained by removing the cofactors THDP and divalent cation through dialysis for 24 hours against Tris–HCl buffer pH 7.5 containing 10 mM EDTA. After removing EDTA by dialysis, different divalent cations were tested as possible cofactors in the transketolase reaction using Assay I and 1.25 mM X5-P and R5-P, respectively.

The effect of metal ions and EDTA, ATP or ADP on TKT activity was measured under standard conditions (Assay I) in the presence of Ca^2+^, Co^2+^, Cu^2+^, Mg^2^, Mn^2+^ and Ni^2+^ at 1 mM final concentration in the reaction mixture. The remaining percentage activities were determined by comparison with no metal ion added. To investigate the effect of EDTA, EDTA salt solution was incubated with TKT for 4 minutes. The measurement was done according to standard assay conditions with 1 mM EDTA final concentration in 1 ml reaction mixture. To study the thermal stability of the TKT proteins, the assay mixture described above was prepared in 1.5 ml reaction tubes and incubated for up to 2 h at 30-80°C. Samples were taken periodically and the residual enzyme activity was measured under standard conditions (Assay I) in a separate reaction mixture.

The TKT activity in the direction of E4-P and X5-P from F6-P + GAP was done by Assay II, a modified version of a previously described assay [31] using the auxiliary enzymes Erythrose-4-phosphate dehydrogenase (E4PDH) from *E. coli* to detect E4-P from the conversion of F6-P and GAP. The oxidation of NADH was followed setting 1 mmol NADH oxidized equivalent to 1 mmol X5-P consumed. The standard reaction mixture (final volume 1 ml) contained 50 mM Tris–HCl buffer (pH 7.5), 0.25 mM NAD^+^, 2 mM Mn_2_Cl, 1 mM dithiothreitol (DTT) 2 U/ml E4PDH and purified TKT protein which was preheated for 3 min at 55°C. NAD^+^ oxidation (ϵ_340nm_ = 6.22 mM^–1^ cm^–1^) was followed at 340 nm on a Shimadzu UV1700 spectrophotometer. The reaction was initiated by the addition of GAP or R5-P respectively (final concentration varied between 0.05 - 10 mM).

Hydroxypyruvate (HP) activity (Assay III) was measured by recording the oxidation rate of the α-carbanion intermediate in the presence of ferricyanide according to the method of Joshi and coworkers (2008) [32]. The reaction mixture in 1.0 ml contained 50 mM glycyl-glycine buffer (pH 7.6), 2 mM manganese chloride, 0.2 mM THDP, 0.5 mM potassium ferricyanide, 3 mM F6-P/HP and 0.24 mg enzyme protein. The reaction was initiated by the addition of enzyme and the reduction of ferricyanide was monitored at 420 nm using UV-1700 PC spectrophotometer (Shimadzu, Japan).

DHAS activity was assayed (Assay IV), depending on the purpose of the experiment, by one of three methods described previously [23,27], with several modifications. For routine assay and to test the effects of glycoaldehyde acceptors on DHAS activity, the activity was measured by a modification of the method of Kato et al. [27] (method A). The reaction mixture (1 ml) contained 50 mmol of standardbuffer (pH 7.0), 0.5 mmol of X5-P, 5 mmol of MgCl_2_, 0.5 mmol of THDP, 0.16 mmol of NADH, 62.6 U TPI (from baker’s yeast; Sigma Chemical Co.), 0.26 U of a GPD (from rabbit muscle; Sigma), and cell extracts.

To test the effect of glyceraldehyde donors on DHAS activity, the activity was assayed by a method based on the system described by Waits and Quayle [23] (method B). The reaction mixture of method B was the same as that for method A except that the mixture (1 ml) contained 1 mmol ATP and 0.23 U of glycerokinase (from *Candida mycoderma*; Sigma) instead of TPI. The mixtures for methods A and B were incubated for 90 s to determine endogenous activity. The reaction was started by the addition of 1 mmol of formaldehyde, and the reduction in absorbance at 340 nm (ϵ_340 nm_ = 6.22 mM^–1^ cm^–1^) was measured between 75 and 105 s after addition of formaldehyde. One unit of enzyme activity was defined as the amount of enzyme required oxidizing 1 mmol of NADH per min.

### Computational analysis

Sequence comparisons were carried out with protein sequences obtained from the NCBI database (http://www.ncbi.nlm.nih.gov), the sequence alignment of the *B. methanolicus* MGA3 TKT proteins and other TKT was done using CLUSTALW [64] and formatted with Box Shade.

## Abbreviations

ADP: Adenosine diphosphate; ATP: Adenosine triphosphate; Bme: *B. methanolicus*; C: Chromosomal; DHA: Dihydroxyacetone; DHAP: Dihydroxyacetone phosphate; DHAS: Dihydroxyacetone synthase; DTT: Dithiothreitol; DXP: 1-deoxy-D-xylulose 5-phosphate Synthase; E4-P: Erythrose 4-phosphate; Eco: *E. coli*; EDTA: Ethylenediaminetetraacetic acid; F6-P: Fructose 6-phosphate; FBA: Fructose 1,6-bisphosphate aldolase; FADH: Formaldehyde dehydrogenase; FBP: Fructose 1,6-bisphosphate; FBPase: Fructose 1,6-bisphosphatase; GAP: Glyceraldehyde phosphate; GlpX: Fructose bisphosphatase; HP: Hydroxypyruvat; HPS: 3-hexulose-6-phosphate synthase; MDH: Methanol dehydrogenase; P: Plasmid; PPP: Pentose phosphate pathway; PHI: 6-phospho-3-hexuloisomerase; R5-P: Ribose 5-phosphate; RPE: Ribulose 5-phosphate 3-epimerase; RPI: Ribose 5-phosphate isomerase; Ru5-P: Ribulose 5-phosphate; RuMP: Ribulose monophosphate; S7-P: Sedoheptulose 7-phosphate; SBA: Sedoheptulose 1,7-bisphosphate aldolase; SBPase: Sedoheptulose 1,7-bisphosphatase; TA: Transaldolase; ThDP: Thiamine diphosphate; TKT: Transketolase; X5-P: Xylulose 5-phosphate.

## Authors' contribution

VFW, BM, JS and TB designed the experiments. BM and JS conducted the experiments, analysed the results, and wrote the manuscript. VFW and TB reviewed and revised the manuscript. All authors read and approved the final manuscript.
